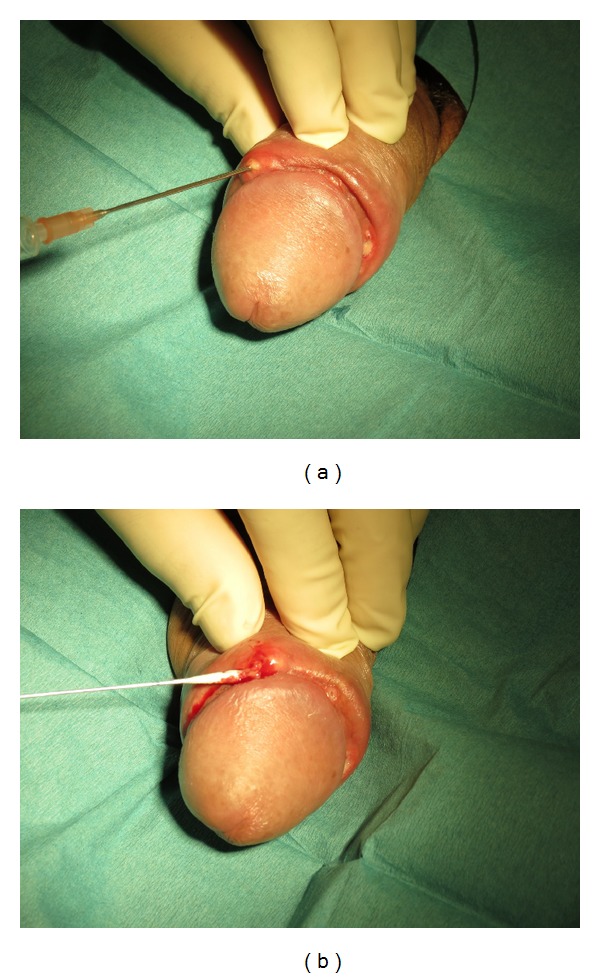# Circular Abscess Formation of the Inner Preputial Leaf as a Complication of a Penile Mondor's Disease: The First Case Report

**DOI:** 10.1155/2014/275752

**Published:** 2014-04-27

**Authors:** Johann Jakob Wendler, Daniel Schindele, Daniel Baumunk, Uwe-Bernd Liehr, Markus Porsch, Martin Schostak

**Affiliations:** Department of Urology and Paediatric Urology, Medical Faculty of the University of Magdeburg, Leipziger Straße 44, 39120 Magdeburg, Germany

## Abstract

*Introduction.* Mondor's disease of the penis is an uncommon condition characterized by thrombosis or thrombophlebitis involving the superficial dorsal veins. An accompanied lymphangitis is discussed. There is typical self-limiting clinical course. *Case Presentation.* This paper firstly reports a secondary abscess formation of the preputial leaf two weeks after penile Mondor's disease and subcutaneous lymphangitis as complication of excessive sexual intercourse of a 44-year-old man. Sexual transmitted diseases could be excluded. Lesions healed up completely under abscess drainage, antibiotic, and anti-inflammatory medication. *Conclusion.* Previous reports in the literature include several entities of the penile Mondor's disease. Our patient is very unusual in that he presented with a secondary preputial abscess formation due to superficial thrombophlebitis, subcutaneous lymphangitis, and local bacterial colonisation. Abscess drainage plus antiphlogistic and antibiotic medication is the treatment of choice.

## 1. Introduction

Penile Mondor's disease of the penis is an uncommon condition characterized by thrombosis, for example, thrombophlebitis involving the superficial dorsal veins. An accompanied lymphangitis is discussed [1]. Several cases as well as duplex sonographic and magnetic resonance imaging findings have been published [2–4]. Historical review of that disease as well as anatomical and physiological relations of the penile venous drainage has been well described [3, 5]. Causes of the penile Mondor's disease are multifaceted: excessive sexual activity, prolonged erection, vacuum erection device, priapism or penile strangulation, penile trauma or injections, urogenital infections, pelvic surgery and prostate biopsy, haematologic diseases, thrombophilia, and paraneoplastic syndrome. Although it has a short, self-limiting benign course, patients experience a firm, nodular, and string-like induration in the coronal sulcus of the penis and along the penile superficial dorsal vein, which is often painful and anxiety-provoking. We report the first case of a penile abscess formation of the preputial leaf as a complication of a penile Mondor's disease and subcutaneous lymphangitis.

## 2. Case Presentation

**Figure 1:**
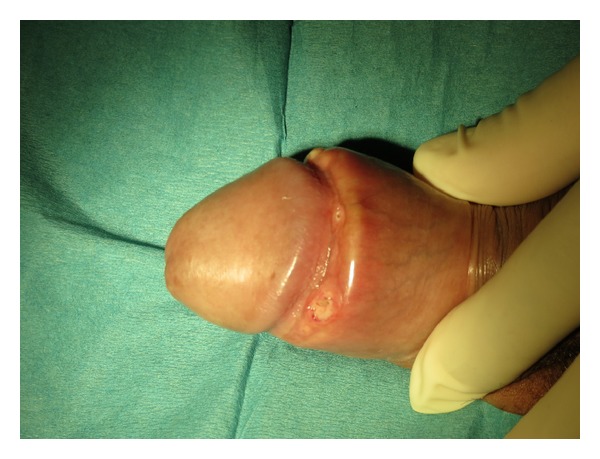


A 44-year-old man presented at our emergency room with a slight painful, subcutaneous, and indurated swelling at the penile dorsum and an oedematous preputium since 2 days. He told of excessive sexual activity without using a condom and fellatio with his wife but denied any penile trauma or manipulations, history of any disease, operations, allergies, or medication. The laboratory and physical examination showed no further pathological findings. B-mode and color Doppler ultrasound indicated an edema of the dorsal penile shaft skin and preputium and the suspicion of a thrombosis of the superficial dorsal penile vein. Cooling, sexual abstinence, and oral medication with ciprofloxacin, diclofenac, and pantoprazole were prescribed. Six days later, he returned because of a progressive swelling of the penile foreskin with a painless pea-sized nodule lump under the inner preputial leaf without any visible superficial lesion. Since two days he received a changed antibiosis to cefixime and doxycycline from his resident physician with doubtful suspicion of cavernitis. No further intervention and the continuation of the aforementioned therapy were recommended. Three days later, he presented an annular abscess formation with partial spontaneous abscess rupture and flush of the inner preputial leaf (Figure 1). There were eight pea- to pinpoint-sized abscesses: six of them were ulcerative after spontaneous perforation; the two remaining nodular abscess formations (Figure 1; Figure 2(a)) were incised with a needle (Figure 2(a)) followed by smears tests (Figure 2(b)), local disinfection, and continuation of the current antibiotic therapy. Three days later, the reexamination showed an improvement but syphilitic-like defects. The smear test was negative for bacterial infection. The dermatological consultant diagnosed a lymphangitis and thrombophlebitis coronarius glandis. The serological analysis was negative for HIV, syphilis, and gonorrhea but positive for mycoplasma pneumoniae (IgG 48.2 U; IgA 11.5; IgM negative). Local disinfection and the continuation of the current medication with cefixime, doxycycline, and diclofenac were recommended. After four more weeks physical and sonographic reexaminations showed a complete healing up of the penile lesions.

## 3. Discussion

Penile Mondor's disease is a rare entity [6]. There seems to be an underestimated incidence because it is a self-limiting process with recanalisation of the vein after one to two months, rare doctor's consultations, and often incorrect diagnoses [7]. The etiology is still controversial. Comparable to our case, excessive sexual activity, prolonged sexual abstinence, and sitting job position can be causally, respectively, risk factors [8]. Erection increasing aid measures that are able to provoke penile Mondor's disease were negated by our patient but cannot be excluded. For idiopathic cases thrombophilia can be considered risk factors [9] whereas our patient had no history of thrombosis and no abnormal laboratory findings. Our patient is very unusual in that he presented with a secondary preputial abscess formation. To the best of our knowledge this complication has not been published before. Similar diseases such as streptococcal pyoderma of the penis [10], penile pyoderma gangrenosum [11], syphilis [12], and other sexual transmitted disease (STD) associated with ulcerous bacterial balanitis [13], balanitis gangrenosa, or erosive circinata [14] could be excluded as differential diagnoses. Various treatment modalities such as antibiotics, anticoagulant drugs, anti-inflammatory agents, and local heparin creams have been proposed in the literature. Penile magnetic resonance (MR) imaging or pelviperineal MR angiography was not performed which could confirm the diagnosis and better investigate the real extension of thrombosis and abscess formation [2]. Although diagnosed lately, the initial commenced and general recommended medicamentous therapy as well as the abscess drainage resulted in a complete healing up with no functional or cosmetical residues in the case of our patient. In conclusion we report the first case of penile abscess formation of the preputial leaf as a complication of a penile Mondor's disease and subcutaneous lymphangitis with bacterial colonisation after excessive sexual intercourse.

## Figures and Tables

**Figure 2:**